# Hospital Meetings

**Published:** 1901-04-27

**Authors:** 


### HOSPITAL MEETINGS.

### METROPOLITAN HOSPITAL FESTIVAL DINNER.

Mr. Henry Allhusen, M.P., presided at the festival dinner of the Metropolitan Hospital, held at the Hôtel Métropole, on the 22nd inst., to which some 150 guests sat down. After the customary loyal toasts had been duly honoured, the chairman proposed "Prosperity to the Hospital." He had lately, he said, been elected to Parliament by a large majority of the most intelligent people of Hackney—(laughter)—and it was only to be expected therefore that he should have a good deal of knowledge of the needs of the district in which that hospital was placed, and he knew that it was regarded as a godsend by the poor people of that neighbourhood. He was glad that in his capacity of prospective chairman of that gathering he had made his appeal for support before Sir Michael Hicks-Beach had announced the increase in the income-tax, for it was his experience that it was not the war funds, but war taxes, that militated against charitable appeals. The public had responded to the appeal made in a very creditable manner and a substantial sum had been raised—though perhaps not so large a sum as the committee desired. But committees never were satisfied; he had never known a well-regulated hospital or a well-constituted committee that was satisfied with the amount of money raised for the object they had at heart. One of the things they had to congratulate themselves upon was that the executors of the late Mr. Zuntz had allocated to them £1,000 a year for ten years, and this after the most careful examination into their methods and management. The work of the hospital had enormously increased; last year they had nearly a thousand in-patients and over 33,000 out-patients. The hospital was in a very poor neighbourhood; in fact, within a radius of two miles, in which they were practically the only institution of the kind, there were living half a million of the poorest people in London. He thought that, considering the effect of the work they were doing, they ought to be spared the burden of local rates and taxes. They paid £177 per annum, and while that might not sound a very large sum, they would see what it meant more if they looked at it from the other point of view, and considered what a handsome annual subscription such a sum would make. It was quite true that the hospital was a ground landlord. They possessed the freehold of a couple of acres of land, and if the hospital were large enough for the work to be done it would be the duty of the committee to sell that land and apply the proceeds to their current needs. But none knew better than the committee that the building was not large enough, and they hoped the time was not far distant when they would be able to extend over some part at any rate of that two acres. He felt constrained to congratulate the committee on the excellence of their secretary, Mr. Byers. He had been greatly impressed with his energy, perseverance, and earnestness on behalf of the hospital, than which he was sure none was better served by its secretary. The toast was coupled with the name of one of their treasurers, Mr. Leopold de Rothschild, and he felt inclined to say, "Happy is the hospital that has a Rothschild for its treasurer!" for it was a name known for its generosity throughout the length and breadth of the land.

Mr. Leopold de Rothschild responded, and reviewed the history of the institution. The Zuntz bequest, he said, would not only be of immense benefit to them, but it was a certificate of merit as well, for the trustees had visited every hospital and dispensary before making their awards and had gone into everything from the top floor to the basement. Another proof that they were worthy of consideration was that the Prince of Wales' Fund gave them a grant, and had increased it year by year. He warmly commended the excellence of the arrangements by which poor Jews who were ministered to here for their bodily ailments had also their religious wants attended to in the most considerate manner, their food being carefully prepared according to their rites. In conclusion, he proposed the chairman's health.

This having been briefly responded to, Sir Francis Jeune proposed "The Committee of Management," which was replied to by the chairman of the hospital, Mr. C. J. Thomas.

The Secretary then announced the amounts collected on the various lists, which totalled £2,958, the Chairman's list amounting to £1,407, closely followed by that of Mr. Franklin (who was so successful last year) with £1,058.

The toast of "The Hospital Staff" was proposed by the Rev. E. A. B. Sanders, and that of "The Visitors" by Mr. Flower, M.P.

The Meistersingers' Orchestra played during the dinner, and the toasts were interspersed by well-rendered songs.

